# Paradoxes in Dentistry

**Published:** 1870-01

**Authors:** 


AMERICAN JOURNAL

OF

DENTAL SCIENCE.

Vol. III. THIRD SERIES--JANUARY 1870. No. 9.

### ARTICLE I.

### *Paradoxes in Dentistry.*

By Professor Austen.

Truth is often made forcible by presentation in the seemingly contradictory form of the paradox. We shall thus present an important point, in connection with the adaptation of the Base Plate, which is often disregarded and perhaps as often, either not known, or not acknowledged.

It often happens that, of several plates for the same mouth the *most* perfect fit is the *least* accurate. In illustration of which, take one case of swaged plate and one case of rubber plate.

In the latter case—greatest care is taken to have the plaster impression perfect and the model without a defect; and yet the piece, made with every precaution drops under use and fails to give satisfaction. An old fogey rival who knows perhaps little about "these new fangled plaster impressions," takes an old fashioned wax impression (possibly none of the best) and his piece fits perfectly, to the great detriment of modern dentistry, and the college trained D.D.S.

In the former case—we make our dies, with such regard to their composition, as shall prevent any possible shrinkage of the metal; and the use of such a number, as shall insure to the plate the full size and exact shape of the model. Agan we fail; and our rival, using the old-timed zinc die, (and one only) succeeds to our grevious discomfiture. Such failures are traceable to causes which it may be profitable to look into.

*First*, the tendency to abandon old methods and materials. Wax, plaster, and gutta percha are each indispensible as impression materials, and he greatly cripples his resources who uses either exclusively. Each takes a different kind of impression, most useful in its own class of cases—wax, the oldest, equally as useful as gutta percha, the latest; and both adapted to a large number of cases, for which plaster, apparently the most accurate of all, is unsuited. Old fashioned zinc not only must not be set aside, because of its contraction; but, for this very reason, is in many cases to be selected. The discovery of new materials does not render old ones useless, but makes it necessary to discriminate the cases which call for one over the other. This leads to the consideration of the

*Second*, and most fruitful source of failure—*routine practice.* By this we mean the more or less exclusive adoption of materials or operations, without sufficient inquiry into the special requirements of the case in hand and without full knowledge of the various resources of dentitsry as adapted thereto. Hence, one will use no impression material but wax, another only plaster, and neither will touch gutta-percha. This one uses zinc and lead, that one only fusible metal, whilst the third prides himself upon the scientific combination of fusible metal dies with type-metal counters. Dr. A. scorns anything but a gold plate; Dr. B. thinks rubber meets all demands; Dr. C. has faith in the old tin base; and Dr. D. hopes much from aluminum, as superseding all other materials.

This tendency to run unthinkingly in one groove, whether of one's own or another's making, is fatal to progress. However much practice may develop the utility of any one material, it can never compensate the advantage of having several, each supplying the deficiencies of the others.

The force required in the use of wax, gives an impression of the mucous membrane in such state of compression as is absolutely necessary in certain cases; hence, in all such, the failure of plaster for rubber work. Again, the fine lines of a plaster impression are faithfully copied in rubber; but they are of no value for a swaged plate, yet plaster may be the best for other reasons.

Gutta percha, for general use, has an objectionable contraction (which, however, may be reasonably controlled); yet this very contraction, in connection with the minute detail of its lines, makes it a most valuable material for rubber work, in lower and full-upper cases.

The contraction of zinc injures the theoretical accuracy of a plate, but often greatly improves its practical efficacy. This contraction we may lessen by a plaster impression, or we may increase it by a gutta percha impression. In case *no* contraction is admissible, and a swaged plate is used, recourse is had to dies which have less contraction than zinc. Tin has less, type-metal still less and that of fusible metal is almost inappreciable. These are all useful in their place, but no system of swaging is perfect, which fails to recognize the distinctive advantages of zinc, due to the very property, which is often urged as an argument against its use. If this be understood, then experiments in metals, which shall give greatest hardness with least contraction, become very instructive and useful.

The character of the mucous membrane, in the special case in hand, is habitually disregarded. Yet upon it mainly depends the selection of the impression material. The selection is also dependent on the kind of base plate. Hence, whatever evidence of perfection an impression may give to the eye; judgement cannot be passed until the practical firmness of its adaptation has tested, not only the general accuracy of the manipulation, but the suitableness of the material selected.

The principles of true science teach a wise selection from abundant resources. We may reasonably hope that this will characterize dental mechanism when the skilled mechanician ceases to be the mere instrument of the unthinking or less informed dental surgeon. By which we mean to say, that he who meets the patieut must be thoroughly familiar with the relative merits and qualities of laboratory material; or he who does the work should have opportunity to decide upon the appropriateness of their selection.

Dental surgery and dental mechanism may both be practiced by the same person, or each in its own completeness by one person. But to practice all of the first and part of the second, in the way it is usually done, gives none of the advantages which accrue from the pursuit of a specialty, and renders impossible that adaptation of varied resources to particular cases which characterizes the perfect mechanician. Good dental surgeons are rare, good dental mechanicians are still more rare; may not one reason be found in this unwise division of the mechanician's duties.

We propose in the next paper to illustrate another dental paradox, to wit; that the greatest improvement in modern dentistry has proved its greatest curse. Vulcanite has added vastly to the resources of the dental mechanician; but it were better, as matters now stand, had this material never passed beyond the limit of combs, buttons, *et id omne genus.*

---

